# There is no correlation between a delayed gastric conduit emptying and the occurrence of an anastomotic leakage after Ivor-Lewis esophagectomy

**DOI:** 10.1007/s00464-021-08962-5

**Published:** 2022-01-03

**Authors:** Benjamin Babic, Lars Mortimer Schiffmann, Hans Friedrich Fuchs, Dolores Thea Mueller, Thomas Schmidt, Christoph Mallmann, Laura Mielke, Antonia Frebel, Petra Schiller, Marc Bludau, Seung-Hun Chon, Wolfgang Schroeder, Christiane Josephine Bruns

**Affiliations:** 1 Department of General, Visceral, Cancer and Transplant Surgery, University of Cologne, Kerpener Strasse 62, 50937 Cologne, Germany; 2 Faculty of Medicine, Institute of Medical Statistics and Computational Biology, University of Cologne, Cologne, Germany

**Keywords:** DGCE, Esophagectomy, Anastomotic leakage, ECCG, Esophageal cancer, Esophagogastric junction cancer

## Abstract

**Introduction:**

Esophagectomy is the gold standard in the surgical therapy of esophageal cancer. It is either performed thoracoabdominal with a intrathoracic anastomosis or in proximal cancers with a three-incision esophagectomy and cervical reconstruction. Delayed gastric conduit emptying (DGCE) is the most common functional postoperative disorder after Ivor-Lewis esophagectomy (IL). Pneumonia is significantly more often in patients with DGCE. It remains unclear if DGCE anastomotic leakage (AL) is associated. Aim of our study is to analyze, if AL is more likely to happen in patients with a DGCE.

**Patients and methods:**

816 patients were included. All patients have had an IL due to esophageal/esophagogastric-junction cancer between 2013 and 2018 in our center. Intrathoracic esophagogastric end-to-side anastomosis was performed with a circular stapling device. The collective has been divided in two groups depending on the occurrence of DGCE. The diagnosis DGCE was determined by clinical and radiologic criteria in accordance with current international expert consensus.

**Results:**

27.7% of all patients suffered from DGCE postoperatively. Female patients had a significantly higher chance to suffer from DGCE than male patients (34.4% vs. 26.2% vs., *p* = 0.040). Pneumonia was more common in patients with DGCE (13.7% vs. 8.5%, *p* = 0.025), furthermore hospitalization was longer in DGCE patients (median 17 days vs. 14d, *p* < 0.001). There was no difference in the rate of type II anastomotic leakage, (5.8% in both groups DGCE). All patients with ECCG type II AL (*n* = 47; 5.8%) were treated successfully by endoluminal/endoscopic therapy. The subgroup analysis showed that ASA ≥ III (7.6% vs. 4.4%, *p* = 0.05) and the histology squamous cell carcinoma (9.8% vs. 4.7%, *p* = 0.01) were independent risk factors for the occurrence of an AL.

**Conclusion:**

Our study confirms that DGCE after IL is a common finding in a standardized collective of patients in a high-volume center. This functional disorder is associated with a higher rate of pneumonia and a prolonged hospital stay. Still, there is no association between DGCE and the occurrence of an AL after esophagectomy. The hypothesis, that an DGCE results in a higher pressure on the anastomosis and therefore to an AL in consequence, can be refuted. DGCE is not a pathogenetic factor for an AL.

Transthoracic en-bloc esophagectomy is the gold standard in the surgical treatment for esophageal cancer and is often performed after neoadjuvant treatment [1–3]. Most commonly reconstruction is performed by a gastric pull-up and a high intrathoracic esophagogastric anastomosis [Ivor-Lewis esophagectomy (IL-OE)] [4]. A three stage esophagectomy with a cervical anastomosis is well-accepted too, particularly in proximal esophageal cancers (McKeown esophagectomy) [5]. This operative procedure is associated with a postoperative morbidity up to 60% and mortality up to 5% [6–8]. Centralisation to high-volume centers, a constant improvement of surgical technique, operation time and a reduction of access related trauma have decreased morbidity and mortality over the past decades [9–13]. Still, one of the most common postoperative complications after IL-OE is a delayed emptying of the gastric conduit (DGCE). The incidence is reported between 2.2 and 47% in a systematic review [14]. A pyloric dysfunction and a reduced gastric motility as a result of the obligatory dissection of vagal nerves, as well as the altered anatomy contribute to DGCE as unswayable factors. It is unresolved if and how much surgery related factors such as conduit size or hiatoplasty impact the risk for occurrence of an DGCE, even though a narrower conduit seems to be favourable to a reconstruction than using the stomach as a whole [15–19].

Certainly, DGCE contributes to short and long-term postoperative morbidity [15, 20]. It is strongly associated with pulmonary complications and a higher risk of prolonged ICU and prolonged hospital stay. Furthermore, it can lead to malnutrition and a reduced quality of life [21–24]. It is not clearly resolved if DGCE is an independent risk factor for anastomotic leakage (AL) and thereby contributing to one of the most threatening complications post esophagectomy, even though there seems to be a tendency towards an increased risk for AL [14, 25]. Several mechanisms explaining how DGCE could contribute to AL are possible. A dilatation of the gastric tube might reduce the microcirculation of the gastric tube and thereby compromise anastomotic healing. Secondly, a dilatation of the gastric tube produces mechanical force on the anastomosis followed by an insufficient healing or a technical failure of the stapled anastomosis. Suspecting DGCE to increase the risk of AL impedes surgeon’s confidence to initiate transition to normal diet in their patients post esophagectomy. This work tests the hypothesis that DGCE represents a risk factor for type II anastomotic leakage by analysing a large cohort of over 800 consecutive patients that underwent Ivor-Lewis esophagectomy for esophageal cancer in a European high volume center.

## Methods

### Study design

A total of 816 patients that underwent transthoracic esophagectomy for esophageal cancer at the Department of General-, Visceral- and Cancer Surgery, University of Cologne, between 2013 and 2018 were included in the study. Data were retrospectively analysed from a prospectively maintained database. The study was approved by the local Ethics Committee of the University of Cologne.

### Operative procedure

A transthoracic esophagectomy with two field lymphadenectomy was performed in all cases. Reconstruction of the intestinal passage was done in all cases with a gastric tube and a high intrathoracic esophagogastrostomy [Ivor-Lewis procedure (IL-OE)]. The detailed technique has been previously described [26]. In brief, a laparoscopic gastrolysis with the creation of a gastric conduit not exceeding 4 cm in width was performed. Esophagectomy, including a bilateral vagotomy and gastric pull-up were performed via thoracotomy. Pyloric drainage procedures such as pyloroplasty e.g. were not part of the operative procedure. All patients received a nasogastric tube until the 2nd postoperative day. Oral intake was started with clear fluids on the 6th postoperative day.

### Clinical parameters and postoperative procedures

Patients were divided into two groups: Group I with DGCE and Group II without DGCE. The diagnosis delayed gastric emptying was determined by clinical (reflux) or radiologic criteria (chest X-ray) in accordance with current international expert consensus. The accepted definition was an output of more than 500 mL over the diurnal nasogastric tube measured on the morning of postoperative day 5 or later or more than 100% increased gastric tube width on a frontal chest x-ray projection together with the presence of an air-fluid level. Clinical symptoms were early satiety, vomiting, nausea and/or regurgitation [27, 28]. Treatment of delayed gastric conduit emptying as a result of pyloric dysfunction was performed endoscopically. Dilatation was performed using a 30 mm esophageal achalasia balloon dilator (e.g., Boston Scientific, Ireland), with a slowly increasing pressure of the balloon under endoscopic control. After intubation of the pylorus, the 30-mm achalasia balloon was inflated to a maximum pressure of 137 kPa for a period of 2–3 min under maximum pressure. After completion the successful dilatation of the pylorus was endoscopically verified [29]. Each anastomotic leakage was classified by an experienced endoscopic surgeon according to the Esophagectomy Complications Consensus Group (ECCG) classification and based on endoscopic and clinical findings during the postoperative course. Type II leakage is defined as a situation requiring interventional but not surgical therapy, such as interventional radiology drainage or endoscopic therapy of the defect [27]. In our series endoscopic treatment of anastomotic leakage ECCG type II was performed either with endoluminal vacuum sponge therapy, stent therapy or both. Patients with leakage that underwent conservative treatment only (e.g., dietery modification, medication: ECCG AL type I) or required operative revision (ECCG AL type III) were excluded from the study.

### Study endpoints

The two groups (DGCE vs. no DGCE) were compared focusing on the rate of postoperative complications and especially on anastomotic leakage.

### Statistical analysis

Statistical significance was determined using the Mann–Whitney test for continuous variables and the *χ*^2^ test or Fisher’s exact test for categorical variables. A *p* value < 0.05 was considered statistically significant. There was no adjustment for multiple testing. Multivariate analysis using a logistic regression analysis estimated the association between endpoints. All statistical analysis was done in SPSS for Windows v25.0 or higher (SPSS Inc., Chicago, IL).

## Results

816 patients were included in the study. A total of 226 (27.7%) patients were diagnosed with postoperative DGCE. Patients with postoperative DGCE had a significantly higher rate of postoperative pneumonia (13.7% vs. 8.5%, *p* = 0.025) and a prolonged hospital stay (median days 17 vs. 14, *p* < 0.001). The risk of postoperative DGCE was significantly higher in female compared to male patients (34.4% vs. 26.2% vs., *p* = 0.040). Table 1 depicts patient characteristics of patients with and without DGCE.

**Table 1:** Characteristics of study collective stratified for the diagnosis of postoperative delayed gastric conduit emptying.1

	Total	DGCE			
		Yes		No	
	*n*	*n*	%	*n*	%
Included patients	816	226	27.7	590	72.3
*Sex*					
Male	665	174	26.2	491	73.8
Female	151	52	34.4	99	65.6
*Age*					
< 65 years	460	129	28.0	331	72.0
≥ 65 years	356	97	27.2	259	72.8
*ASA Score*					
I	27	8	29.6	19	70.4
II	448	116	25.9	332	74.1
III	332	98	29.5	234	70.5
IV	9	4	44.4	5	55.6
*Histology*					
AC	642	183	28.5	459	71.5
SCC	174	43	24.7	131	75.3
*Anastomotic leakage type II*					
No	769	213	27.7	556	72.3
Yes	47	13	27.7	34	72.3
*Dindo-Clavien Score*					
≤ IIIA	721	204	28.3	517	71.7
IIIB–IVB	84	21	25.0	63.0	75.0
V	11	1	9.1	10	90.9
*Postoperative pneumonia*					
Yes	46	17	37.0	29	63.0
No	735	195	26.5	540	73.5
*Length of hospital stay (days)*					
Median/IQR	15	17	15–22	14	12–18

*IQR* Interquarantile range

47 patients (5.8%) were diagnosed with postoperative anastomotic leakage ECCG type II. 769 patients out of this selected cohort (94.2%) had no anastomotic leakage. The two patient groups did not differ regarding baseline clinical characteristics like sex, age, ASA and histology. Furthermore, there was no difference in patients that underwent neoadjuvant treatment in both groups. Baseline characteristics for patients with and without anastomotic leakage are depicted in Table 2.

**Table 2:** Characteristics of study cohort stratified for the diagnosis of anastomotic leak

	Total	Anastomotic leak			
		No		Type II	
	*n*	*n*	%	*n*	%
Included patients	816	769	94.2	47	5.8
*Sex*					
Male	655	626	94.1	39	5.9
Female	151	143	94.7	8	5.3
*Age*					
< 65 years	460	433	94.1	27	5.9
≥ 65 years	356	336	94.4	20	5.6
*ASA Score*					
I	27	27	100.0	0	0.0
II	448	427	95.3	21	4.7
III	332	307	92.5	25	7.5
IV	9	8	88.9	1	11.1
*Histology*					
AC	642	612	95.3	30	4.7
SCC	174	157	90.2	17	9.8
*Delayed gastric conduit emptying*					
No	590	556	94.2	34	5.8
Yes	226	213	94.2	13	5.8
*Dindo-Clavien Score*					
≤ IIIA	721	689	95.6	32	4.4
IIIB–IVB	84	73	86.9	11.0	13.1
V	11	7	63.6	4	36.4

To test our hypothesis that patients with DGCE are associated with a higher risk for the occurrence of AL than patients without DGCE after esophagectomy, we investigated both collectives for AL. AL occurred in 5.8% of patients with DGCE and had an identical rate of 5.8% in patients without DGCE, respectively. In consequence, DGCE was not associated with AL Type II in our collective.

We analysed other potential factors which might have a possible impact on the occurrence of anastomotic leakage. First, the American Society of Anaesthesiology (ASA) Score, classifying patients in terms of their perioperative risk with respect to their preoperative condition [30]. We subdivided our patients in one group with patients classified as ASA I or II and compared them to a group of patients classified as ASA III or IV, assessed during preoperative work up by the visiting anaesthesiologist. ASA III and IV patients had a higher risk to suffer from AL (7.6%) than patients with ASA I and II (4.4%, *p*-value 0.05). We were furthermore able to show, that patients with a squamous cell carcinoma (SCC) of the esophagus have a significantly higher risk of AL compared to patients with adenocarcinoma (9.8% vs. 4.7%, *p* = 0.01). R0 resection rates for AC and SCC were comparably high (98% vs. 97%) and therefore not causally associated to the occurrence of DGCE after surgery.

In line with this, as depicted in Table 3, the logistic regression analysis revealed that in our collective the risk to develop AI type II is reduced by around 50% in patients with AC (OR 0.45, 95%-CI 0.24–0.83, *p*-value = 0.011) compared to SCC as well as in patients with ASA score I or II compared to ASA score III or IV (0.55, 95%-CI 0.30–1.00, *p*-value 0.05).

**Table 3:** Logistic regression analysis of clinical parameters associated with anastomotic leakage

Variable	*p*-value	Odds ratio (OR)	95% CI for OR	
			Lower	Upper
Histology (AC vs SCC)	0.011	0.45	0.24	0.83
ASA category (I + II vs III + IV)	0.050	0.55	0.30	1.00
Age category (< 65 vs ≥ 65)	0.615	1.17	0.64	2.14
DGCE (no vs yes)	0.952	1.02	0.52	1.98

*CI* confidence interval; *OR* odds ratio

## Discussion

Our data clearly demonstrate that delayed gastric conduit emptying after Ivor-Lewis esophagectomy is a common functional disorder. DGCE can likely result in high morbidity with increased rates of postoperative pneumonia followed by a prolonged hospital stay. In our collective there was no association between a DGCE and an increased risk for anastomotic leakage. Though this is a retrospective single-center analysis our work has several strengths: (i) we analysed over 800 cases within 5 years from a prospectively maintained database (ii) the operative technique was identical in every case and was highly standardized (iii) our collective is highly representative as associations between DGCE and pneumonia e.g., could be retraced. To our knowledge, this is the largest cohort of patients examined for DGCE after a standardized treatment. With a DGCE rate of 27.7% in our collective the results are well comparable to international data [14]. Since ECCG Type I AL is a rare finding without a clinical or therapeutic relevance and thus without statistical impact they were no subject to this analysis. AL type III was usually diagnosed on the second postoperative day, before oral intake was started and therefore no diagnostic work up regarding DGCE was performed, these cases missed any significant statistical contribution to this study and were, therefore, excluded. Furthermore, patients with AL type III had clear signs of a not sufficient blood supply of the gastric conduit, which explained the cause for anastomotic leakage itself and, therefore, DGCE was not relevant at all.

One simple explanation for differences seen between the association of DGCE and AL compared to previously published results from others is that several publications include patients operated with different surgical techniques (including McKeown procedure, cervical anastomosis) that might have biased the results [25, 31]. Another potential bias might be, that DGCE usually is treated endoscopically, increasing the possibility to detect small, clinically inapparent anastomotic leakages during the endoscopic procedure [29]. A recent report from Hadzijusufovic et al. evaluating the usefulness of a preoperative endoscopic balloon dilatation as a strategy to prevent postoperative DGCE indirectly supports our findings. The authors did not find a significantly decreased rate of anastomotic leakage or early pulmonary complications in their intervention group (preoperatively dilatated pylorus) [32]. Indeed, the latter work demonstrated a significantly lowered rate of DGCE in patients that underwent preoperative dilatation of the pylorus during staging/restaging endoscopy compared to patients who did not receive an endoscopic pneumatic dilatation of the pylorus prior to Ivor-Lewis esophagectomy. Still, a preoperative dilatation usually is performed with a 20 mm balloon, whereas a postoperative dilatation can be performed with a 30 mm balloon, which was initially designed for the treatment of achalasia patients. Thus, a re-dilatation was more often necessary in patients after a 20 mm balloon procedure. Randomized clinical trials that evaluate the interventional option of preoperative dilatation or postoperative medical therapy are currently lacking in the literature and should clearly be considered in the future. At least a partial recovery of the gastric peristalsis has been reported earlier, which might even improve by medical therapy [33–35]. This is in line with reports of a regaining of gastric acidity in the denervated stomach [36]. Surgical perioperative pyloric drainage procedures like pyloromyotomy or pyloroplasty seem to significantly lower the risk of DGCE but without any impact on other morbidities such as AL and/or pulmonary complications, as reported in the meta-analysis by Urschel et al. [37]. In addition as Palmes et al. described surgical pyloric procedures are reported to contribute to a higher rate of bile reflux and esophagitis without having a significant benefit in the early postoperative phase, even though there seems to be a trend towards less early pulmonary complications and a lower rate of anastomotic leakage [38]. This trend is in line with the data published by Hadzijusufovic, as described earlier [32]. This might be the case, since the surgical drainage procedures are more invasive and usually a definitive therapeutic option compared to the endoscopic balloon dilatation, which sometimes even has to be repeated for a successful therapy. In summary, there is no high-quality evidence which supports pre- or perioperative pyloric drainage procedures to significantly contribute to a reduction of postoperative morbidity, even though the rate of DGCE can be reduced. Obviously, the interrelation of DGCE and postoperative morbidity is not finally understood and requires further investigation.

Another issue that has to be addressed is that there remains a small group of patients with long-term complaints associated to DGCE being refractory to repeated pyloric dilatation. This is a very demanding situation and depends on individual clinical properties of each patient. In these patients a pyloric spasm does not seem to be the reason for DGCE, more often it is a hiatal narrowing or a kinking of the gastric conduit, which results in a kind of gastric outlet obstruction. Treatment strategies in our own collective range from conservative prokinetic drug treatment through endoscopic pyloric stenting to surgical revision. More standardization of this clinical problem is further needed to define treatment recommendations. There is a lack of literature for this rare but regularly occurring problem. At least, a consensus about the definition of DGCE found broad acceptance and therefore benefits to a better comparability of international studies [28].

Overall, our work underscores that DGCE has to be considered as a frequent complication after Ivor-Lewis esophagectomy and a reconstruction with a gastric conduit pull-up. It significantly contributes to postoperative morbidity, is primarily associated to an increased rate of pulmonary complications and a prolonged hospitalization. According to our findings DGCE can be seen as its own entity of postoperative morbidity. In the absence of other clinical or endoscopic signs DGCE should not be a direct warning sign for an accompanied anastomotic leakage making it an important finding for treatment pathways of affected patients. Clinical and/or radiologic suspicion of DGCE in the absence of signs of infections does not require urgent endoscopic intervention which might have its own risks. Usually, a drainage with a nasogastric tube resolves most clinical symptoms and gains time for a planned endoscopic therapeutic procedure under controlled and safe circumstances.
